# Neglected Tropical Diseases, Neglected Data Sources, and Neglected Issues

**DOI:** 10.1371/journal.pntd.0000104

**Published:** 2007-11-07

**Authors:** Burton H. Singer, Carol D. Ryff

**Affiliations:** 1 Office of Population Research, Princeton University, Princeton, New Jersey, United States of America; 2 University of Wisconsin, Madison, Wisconsin, United States of America; Swiss Tropical Institute, Switzerland

Given the biomedical roots and disease treatment orientation of the Global Burden of Disease (GBD) framework, as exemplified in the World Bank's 1993 World Development Report, *Investing in Health* [1], it is not surprising that we are at a crossroads where whole categories of diseases and a new journal have the adjective “neglected” in their titles. Some of this neglect might have been avoided, even at the outset, if the authors of the 1993 World Development Report had paid attention to the contents of the 1992 World Development Report, which focused on the environment [2]. In virtually every chapter and chapter summary of the 1992 report, issues of health appear, and disease prevention—i.e., reducing the demand for treatment—is center stage. The imbalance between prevention and treatment manifests itself in unsustainable contemporary programs where, for example, a strong case is put forth on behalf of drug packages for treating/de-worming people infected with a range of parasites on the current neglected tropical disease (NTD) list [3], while no mention is made of the fact that clean water and sanitation would prevent re-worming by a considerable list of intestinal parasites and soil-transmitted helminths following the needed de-worming via drugs. We note that hookworm suppression in the southern United States nearly 100 years ago featured both de-worming with drugs and the prevention of re-worming by provision of sanitary facilities [4]. The contemporary “Schistosomiasis Control Initiative” (SCI) [5] is focused on distribution of praziquantel and albendazole for de-worming to improve the lives of infected people and protect children from future disease. However, it is worrying to us that SCI does not have funds to tackle two of the key risk factors, clean water and sanitation, in the priority health statistics list for the GBD program [6],[7]. These examples illustrate the need for bridge building between the water and engineering sectors and the biomedically focused component of the health sector [8],[9]. Indeed, many engineering nongovernmental organizations and private donors are *prioritizing*, not neglecting, clean water and sanitation projects, but unfortunately undertake no follow-up evaluation of health consequences due to their minimal connection to the health sector [8],[9].

The above preamble and a review of the measurement issues discussed by Mathers et al. [7] point to multiple areas of neglect, and thereby opportunities for improvement in the future of the GBD program. We focus on three areas, namely (i) comorbidity, (ii) the integration of human and animal health data, and (iii) enhancing data systems via linkages to the World Trade Organization (WTO). Our invitation from *PLoS Neglected Tropical Diseases* to write a viewpoint based on Mathers et al. [7] encouraged us to critically evaluate formulations of the global burden as expressed in disability-adjusted life years (DALYs) lost, and a variety of measurement problems connected with the current GBD framework. Extensive critiques of the extant framework have already appeared, many of them referenced in [7]. Rather than repeating these critiques, we instead call for far more extensive revision of outcome measures and of the entire GBD framework, based on the agenda we put forth below.

## Comorbidity Is Pervasive

Beginning with the list of NTDs that defines the focus of this journal, we find that polyparasitism is the rule rather than the exception in many tropical communities. In an in-depth study of a village in the region of Man, western Côte d'Ivoire, Keiser et al. [10] found that two-thirds of the population harbored three or more parasites concurrently from a list of 12 parasites investigated that included four helminths (*Ascaris lumbricoides,* hookworm, *Schistosoma mansoni*, and *Trichuris trichiura*) and eight intestinal protozoa, the most common being *Entamoeba coli, Blastocystis hominis, Endolimax nana*, and *Iodamoeba butschlii*. It has also been found that among school-aged children across 56 communities in the region of Man, 19% of children had coinfections with *S. mansoni* and hookworm [11]. Some of the schools had coinfection rates with these two parasites exceeding 50%. It is important to note that these reports exclude malaria, which is also common in the same communities. Further, coinfection with malaria and HIV has recently been highlighted as a source of increased severity of both these diseases in sub-Saharan Africa, and there is a growing literature focused on coinfection with diverse combinations of helminths, HIV, malaria, and tuberculosis [12].

Emphasizing the “global” in GBD, it is important to note that among elderly persons in the United States and much of Europe, comorbidity in the form of multiple co-occurring chronic conditions is also the norm rather than the exception. In US surveys [13], 61% of women and 47% of men aged 70–79 years report at least two chronic conditions. These figures rise to 70% of women and 53% of men aged 80–89 years. No single form of comorbidity occurs with high frequency, but rather a multiplicity of diverse combinations is observed (e.g., diabetes and osteoarthritis, colon cancer and coronary heart disease, depression and osteoporosis, etc.). The occurrence of this phenomenon in resource-rich countries (and likely in developing countries if we had the data to show it), together with coinfection with NTDs among the most disadvantaged and impoverished peoples, suggests the need to reformulate the notion of “burden of disease” to make it compatible with the realities that many individuals face. If a primary goal of the next generation of GBD data is to assist resource allocation to deal with the disease mix faced by large populations, then ascertainment of heterogeneous forms of comorbidity must be a high priority.

## The Need to Integrate Data on Human and Animal Health

Many newly emerging diseases (e.g., avian influenza), as well as long established members of the NTD list (e.g., Japanese encephalitis) have links to either domestic or wild animals, or both [14]. Indeed, most human infectious diseases have animal origins. These linkages underscore the need for connection between human and veterinary medicine, since animal health plays an important role as a risk factor in the burden of human disease. There are also related policy implications derivable from selected studies of animal-derived human infection (e.g., brucellosis) where vaccination of cattle, for example, is more cost-effective than interventions on humans to prevent new infection [15].

The zoonotic origin of many infectious diseases of humans leads directly to a rationale for combining data collection on human and animal health in the GBD program. The unfortunate separation of human and veterinary medicine throughout the 20th century has, however, resulted in a corresponding separation of the World Organization for Animal Health [16],[17] from the World Health Organization (WHO). Fortunately, study groups formed by WHO are starting to focus on veterinary public health and its linkage to human public health. An important basis for bridge building in a revised framework for GBD would be integration of the “Advanced Veterinary Information System,” established by the Food and Agriculture Organization in 1992 [18] with geo-coded human disease data bases.

## The Need for More and Better Primary Data Collection in the Tropics

Much criticism has been directed at the GBD program to date as a result of its use of a variety of adjustment and imputation schemes on sparse primary data for the purpose of providing “improved and less biased” statistics [6]. Everyone agrees about the pressing need for more extensive high-quality primary data on human disease in tropical countries, but the necessary political will and financial resources to develop and maintain the requisite data bases is missing. This constitutes an even more difficult problem when the integration of human and animal health is incomplete.

A way forward is in progress, but there is pressing need for the international public health community to engage with the potential created by the Sanitary and Phytosanitary (SPS) measures agreement negotiated in the Uruguay Round of the General Agreement on Tariffs and Trade from 1986 to 1992. The central point is that this agreement has been adopted by all member states of the WTO. The SPS surveillance requirements force an integration of human and animal health. Specifically, the provisions [19] require that: “Members shall ensure that their sanitary or phytosanitary measures are based on an assessment, as appropriate to the circumstances, of the risks to human, animal or plant life or health, taking into account risk assessment techniques developed by the relevant international organizations.” Further, “Members shall take into account available scientific evidence; relevant processes and production methods; relevant inspection, sampling and testing methods; prevalence of specific diseases or pests; existence of pest—or disease—free areas; relevant ecological and environmental conditions…” The demanding surveillance requirements of the SPS agreement impose a financial and capacity burden on tropical countries. However, because they are part of a trade agreement, compliance becomes a priority at the highest levels of governments. This agreement has motivated new partnerships between developed and developing countries in the establishment of data systems that, indeed, bridge human and animal health. Although the health issues that require a focus for the SPS agreement do not, by any means, meet the needs of a full GBD system, they nonetheless facilitate the establishment of high-quality primary data platforms in the tropics that can become the basis for later expansion to more complete systems that fulfill the larger public health needs. Thus, establishing much needed improved tropical country primary data systems, with initial revenue streams coming from trade-based incentives, should be viewed as a multistage process. Funding to expand already established, and financially sustainable, integrated human–animal data systems should be far less demanding than the financial requirements of building the full primary data GBD system from the ground up.

## Discussion

The Mathers et al. paper [7] “examines priorities and issues for the next major GBD study, funded by the Bill & Melinda Gates Foundation, and commencing in 2007.” We view the stated proposal as largely fine-tuning of an existing framework rather than an attempt to develop a much needed conceptual and methodological *reformulation* with related data collection activities that would yield a vastly more realistic picture of human disease burden. Seriously addressing comorbidity and the integration of human and animal health would represent a major stride forward in eliminating neglected environmental and sanitary issues, and would concomitantly bring NTDs into greater prominence. Equally important is the potential for establishing long-term revenue steams to finance primary data collection in the tropics by building on trade agreement surveillance requirements. The WTO may be a difficult partner to work with, but there are public health payoffs from a focus on trade-related health issues. While WHO may seem like the more natural institution through which to orchestrate the requisite primary data collection for the next generation of GBD reporting, WHO lacks the clout and basis of support needed at the highest levels of government to secure stable revenue streams needed to sustain such a system. The WTO, on the other hand, is more limited in human disease scope, but because of its focus on trade, it commands greater attention in establishing primary data collection platforms.
